# Predicting pediatric sepsis: bridging the gap between diagnosis and early intervention

**DOI:** 10.1038/s41390-025-04475-2

**Published:** 2025-10-21

**Authors:** Allan M. Joseph, Jennifer M. Kaplan

**Affiliations:** 1 Department of Pediatrics, University of Cincinnati College of Medicine, Cincinnati, OH USA; 2 Division of Critical Care Medicine, Cincinnati Children’s Hospital Medical Center, Cincinnati, OH USA

## Abstract

Early identification of pediatric sepsis is critical but remains challenging. This commentary highlights the potential of predictive modeling to enhance care in the PICU using the Phoenix criteria, while emphasizing the need for external validation and integration into clinical workflows.

Sepsis—life-threatening organ dysfunction related to infection—is a leading cause of morbidity and mortality in children and is often readily apparent to clinicians at the bedside. However, the wide heterogeneity within this syndrome has prompted three decades of efforts to formalize diagnostic criteria for use in research and clinical care. In pediatrics, the recent Phoenix consensus criteria for sepsis advanced this effort by applying machine learning techniques to millions of encounters across the world in order to identify children at risk of dying from infection.^1,2^ The Phoenix criteria, however, were explicitly “not designed for screening children at risk for developing sepsis or early identification of children with suspected sepsis,” though the authors were hopeful that such tools could “lead to early interventions that decrease the morbidity and mortality associated with pediatric sepsis.”^1^

Chanci et al.^3^ attempt to bridge this gap by using electronic medical record data to predict which patients in a pediatric ICU (PICU) will meet the Phoenix criteria in the near future. The authors leverage a unique setup in which a single health system operates two high-acuity PICUs, allowing one unit to serve as a derivation cohort to develop a prediction algorithm, and the other to serve as a validation cohort to test the algorithm’s performance. They identified 2379 encounters meeting Phoenix sepsis criteria in the derivation cohort and fit four different machine-learning algorithms to structured data in the encounter, including the patient’s age, vital signs, lab values, and medications. Notably, the calculated pediatric Sequential Organ Failure Assessment (pSOFA) score was also included in the primary analysis, reflecting diagnostic criteria adapted from the Sepsis-3 definition for adult patients.^4^ They then applied these models to the validation cohort to evaluate model performance, finding excellent performance in all four models led by the Categorical Boosting model. The classic test of discrimination, area under the receiver operating characteristic curve (AUROC), had an excellent value as high as 0.98. The area under the precision recall curve (AUPRC)—which better evaluates discrimination when the outcome of interest is relatively rare—was also as high as 0.83, suggesting the model performed 10 times better than a random prediction given the incidence of sepsis of 8.1% in the validation cohort.^5^

Importantly for bedside clinicians who wish to evaluate the plausibility of a predictive model, the authors report which clinical features drive model predictions. The strongest predictor is the pSOFA score, followed by elements that reflect the patient’s primary diagnosis; most likely, the presence of medications for asthma and seizures reliably differentiates patients without sepsis from patients who have it. Other important features generally reflect developing organ dysfunction and vital sign abnormalities—but taken together, the model could predict the onset of sepsis approximately 19 h prior to Phoenix criteria being fully met.

Despite strong model diagnostics, no predictive tool can be perfect, and evaluation of these tools requires examination of the cases which are misclassified. Here too the models perform well. Patients who are “false positives” are a sicker-than-average group of patients, with three times higher risk of in-hospital mortality than the overall study population (see Supplementary Table S8). Additionally, there is a small group of “false negative” patients who met Phoenix sepsis criteria without identification by the model; however, this group has lower mortality than those patients identified by the predictive models, suggesting that the predictive models are well-targeted to identify those patients who are at the highest risk of mortality related to sepsis.

Thus, Chanci et al. have developed models that can reliably identify patients who are likely to meet Phoenix sepsis criteria in their health system. Moreover, where they err, they do so by over-identifying sick patients and missing relatively low-risk patients. Two important questions that must be answered to move these findings towards widespread clinical application. The first is external validity: whether these models can work in other PICUs with potentially different patient populations. These models were developed in an academic quaternary PICU and then validated in a community tertiary PICU with a presumably different patient population, but there are some important patient populations that may not be reflected in the validation cohort. Most notably, immunocompromised patients such as those who have received solid-organ transplants or have complex oncologic disease (including the receipt of hematopoietic stem cell transplants) may not be represented in the tertiary PICU. These patients, who frequently have pre-existing organ failures and are at high risk of mortality from sepsis, are potentially the highest-value patients in whom to predict the onset of sepsis, but model performance in this group remains unclear, and likely requires further evaluation as models are adapted to new units.

The second question is how to leverage early prediction of sepsis to improve outcomes. Identifying children likely to develop sepsis is only the first step. To translate into reduced mortality and morbidity, this identification must then change the way in which patients are monitored or treated. There are currently no targeted treatments that reduce the risk of progression from infection to sepsis, though an ability to predict sepsis-related organ failure hours in advance would provide a window of opportunity should such therapies be developed. Until then, predictive models may still improve outcomes by enhancing team dynamics and situational awareness. One such example in PICUs relates to the use of structured multidisciplinary efforts to identify patients at high risk of in-hospital cardiac arrest (IHCA) and develop tailored mitigation plans that can be enacted at the first sign of decompensation. Observational studies suggest these interventions can lead to meaningful reductions in the rates of IHCA and mortality,^6,7^ and a stepped-wedge randomized controlled trial (SAMURAI PICU, NCT06553534) is ongoing to confirm these findings. Because automated clinical decision support tools are a key pillar of these interventions, one can imagine how models such as those developed by Chanci et al. could be incorporated into an expanded approach to situational awareness in the PICU. Moreover, the fact that “false positives” in these algorithms are a relatively sick group suggests that these patients may benefit from these plans even if they do not ultimately develop sepsis.

Finally, it is worth noting that this work is developed and validated only in patients already admitted to the PICU, and does not address prediction of sepsis for patients in the emergency department or admitted to an inpatient ward. In particular, patients admitted to general inpatient wards, where the frequency and amount of laboratory and other data collection may be significantly less than that in the PICU, represent a different methodological challenge that remains to be addressed. National efforts in this arena have focused on timely identification and early resuscitation with encouraging results,^8^ but have not yet moved into prediction—which will be challenging given lower incidence rates in this population.

The work by Chanci et al. represents a meaningful step forward in the application of machine learning techniques to improve the care of critically ill children with sepsis. Future research and quality improvement efforts should evaluate the external validity of these models, as well as their incorporation into clinical decision support tools and situational awareness tools so as to translate prediction into changes in care. These questions will ultimately determine the ability of these models—and of the Phoenix sepsis criteria—to meet the goal of reducing the morbidity and mortality associated with pediatric sepsis.
